# The teams of early-career investigators: A qualitative pilot study

**DOI:** 10.1017/cts.2018.335

**Published:** 2019-02-05

**Authors:** Marie K. Norman, Megan E. Hamm, Colleen A. Mayowski, Yael Schenker, Wishwa N. Kapoor

**Affiliations:** 1 Institute for Clinical Research Education, University of Pittsburgh School of Medicine, Pittsburgh, PA, USA; 2 Center for Research on Health Care Data Center, University of Pittsburgh School of Medicine, Pittsburgh, PA, USA; 3 Section of Palliative Care and Medical Ethics, Division of General Internal Medicine, University of Pittsburgh School of Medicine, Pittsburgh, PA, USA

**Keywords:** Education, team science, clinical research training, qualitative methods, science of team science, early-career investigators

## Abstract

*Introduction:* Early team experiences can influence the professional trajectories of early-career investigators profoundly, yet they remain underexplored in the team science literature, which has focused primarily on large, multisite teams led by established researchers. To better understand the unique challenges of teams led by early-career investigators, we conducted a qualitative pilot study.

*Methods:* Interviews were conducted with the principal investigator and members of 5 teams led by KL2 and K12 scholars at the University of Pittsburgh. A code book was developed and thematic analysis was conducted.

*Results:* Seven distinct themes emerged. Interview subjects reported a high level of trust and strong communication patterns on their teams; however, the data also suggested underlying tensions that have the potential to escalate into larger problems if unaddressed.

*Conclusions:* This study yields a deeper understanding of teams led by early-career investigators, which can help us provide appropriately targeted training and support.

## Introduction

Medical research confronts increasingly complex, multicausal health issues that require investigation by interdisciplinary teams [1–4]. Because poorly functioning teams can squander human capital, resources, and time, the science of team science, or SciTS, has emerged to investigate the factors that contribute to team success [5].

SciTS has contributed valuable insights into issues distinctive to science teams and has offered a range of relevant theoretical models and recommendations [4]. However, much of the SciTS literature has focused on the challenges of large, geographically distributed teams, typically led by established researchers. Considerably less is known about the unique challenges of early-career investigators and the teams they lead. Because early-career investigators are often new to team leadership, supported by relatively small training grants, and in the early stages of launching research careers, we would expect their experiences—as well as those of their team members—to be somewhat different than those of senior investigators. Given the importance of early team experiences in shaping the professional trajectories of junior investigators and their teams, we believe these experiences warrant closer scrutiny.

The difference in experiences between groups guided by established leaders versus novice leaders has been explored more thoroughly in other fields, for example, in education [6–8] and the US military [9]; however, it has not been systematically investigated in the context of scientific research. Moreover, as Day *et al.* observe, while the broader literature on leadership has advanced significantly over the past 25 years, there remains a need for more systematic, empirical investigation of leadership as a developmental process [10]. This call is consistent with the contention put forward by Falk-Krzesinski *et al.* [5] that we need to understand the needs of scientists at different career stages in order to develop more appropriate models for team training.

This paper describes the results of a pilot study conducted between February and November 2017 by the Institute for Clinical Research Education at the University of Pittsburgh. In this study, we examined the experiences and challenges of clinical and translational science teams led by early-career investigators. Our goals were to identify factors that contribute to positive and negative team experiences, challenges and sites of tension related to team functioning, and the skills and conditions that contribute to motivated, productive teams. Ultimately, our intention is to use the insights from this research to develop team science training specifically tailored to meet the needs of early-career scientists.

## Methods

We conducted qualitative interviews with the leaders and members of 5 teams led by junior faculty, all of whom were trainees at the Institute for Clinical Research Education at the University of Pittsburgh. We chose team leaders from the Clinical and Translational Science Scholars Program (KL2) and the Comparative Effectiveness Research Scholars Program (K12) because they are mentored early-career investigators with interdisciplinary teams.

None of the team leaders had received formal training in team science through our programs, although a team training workshop was instituted after this research was conducted (Mayowski *et al.*, unpublished data, 2018). Leadership skills are taught in a number of seminars and courses in the School of Medicine, so it is likely that principal investigators (PIs) had at least some leadership training before this research. We are not aware of any team science training that other team members received, although—given the ubiquity of teams and team training in many professional and personal contexts—we cannot rule it out.

We approached KL2 and K12 scholars during a professional development seminar, and explained that we were conducting research to better understand team experiences with the goal of designing effective team training. We clarified that participation was optional, then distributed pen-and-paper surveys which asked scholars to identify their grant funding, describe the size and composition of their teams, and indicate their willingness to participate in the study (Appendix A). Participants were informed that responses were anonymous unless they agreed to be interviewed, in which case we asked for their name and contact information.

We followed up with an email (Appendix B) to the scholars who indicated their willingness to be interviewed, confirming their intention to participate and requesting permission to approach their team members as well.

Eight of 9 scholars agreed to be interviewed. We used purposive sampling to select 5. Our primary inclusion criterion was whether they led teams composed of 6-10 people. Although we recognize that even very small teams can encounter team process and leadership challenges, we wanted to focus on teams that included representation from roles that are common on clinical research teams, including PIs, mentors, collaborators, statisticians, research coordinators, and research assistants. Because very small teams would reduce our chances of getting representation from each of those 6 categories, we excluded teams with under 6 members. We chose 10 as our upper limit, both for logistical feasibility and because it is in keeping with the National Research Council’s range of 2-10 people as the normative size for science teams [4]. We limited the study to 5 teams assuming that they would yield roughly 30 interviews, the number of interviews at which reaching thematic saturation is likely [11]. Interview questions were oriented toward general team dynamics and functioning rather than specific team roles; thus, we determined that role-based thematic saturation was unnecessary in a pilot study.

A qualitative methodologist (M.H.) and a trained qualitative researcher from our Qualitative Evaluation and Stakeholder Engagement Research Service (Qual EASE) conducted 1-hour, semi-structured interviews with the PIs on 5 teams (i.e., the KL2 and K12 scholars who indicated their willingness to participate). After interviewing the PIs, we requested the names and contact information of their team members, then contacted team members to request interviews. Team members were informed that their participation was voluntary, interview data was strictly confidential, and they could opt out without their PI or teammates knowing.

Team members who consented to the interview were interviewed individually, either by phone or in person according to the preference of the participant. Participants were asked a similar set of questions to the PIs. Both PIs and team members were asked to describe their teams and projects, explain their individual and team goals, describe team structure and organization, talk about how the team dealt with challenges and differences of opinion, give examples of when the team had worked well or poorly, and provide general reflections on team dynamics (see Appendices C and D for in-depth interview guides). Questions were adjusted according to the position of the team member, with team formation questions reserved for the PI. Interviews were linked so we could compare perspectives on a given circumstance or event, but in order to preserve anonymity, we did not ask team members about experiences described by other team members. Participants gave consent verbally before interviews were conducted. All research protocols were covered under IRB #0608202.

In addition to the 5 PIs, we approached 31 team members and 17 agreed to be interviewed. The roles of the participants are shown in Table 1. Three to five members of each team participated in the interviews.

**Table 1:** Team roles and number of participants (n=22)

Role	No. of participants
Team Lead/PI	5
Mentor	5
Consultant/Collaborator	5
Research Assistant	4
Statistician	2
Research Coordinator	1

PI = principal investigator.

## Analysis

Data analysis was conducted by a qualitative methodologist (M.H.) with the assistance of qualitative researchers at Qual EASE, following the 6-step process for thematic analysis described by Braun & Clarke [12]. All 22 interviews (5 PIs, 17 team members) were transcribed verbatim. A codebook was inductively developed by Dr Hamm and the other Qual EASE interviewer. The same Qual EASE staff member served as the primary coder for the project. The primary coder and a secondary coder (also from Qual EASE) applied the codebook to 10 common transcripts drawn from 3 of the 5 teams and representing all team roles, achieving a mean Cohen’s κ score of 0.62, indicating substantial agreement. All coding disagreements were adjudicated to full agreement, following which the primary coder independently coded the remaining interviews. Coding was completed in Atlas.ti[13] in order to facilitate analysis. This coding formed the basis of a thematic analysis [12,14], which was conducted on the coded interview segments by Dr Hamm with the input from the primary coder. Coded data were examined in order to look for themes, or patterns, in participant responses. These themes were discussed and refined with other team members who had not conducted or coded the interviews, but who had content expertise in team science, as a form of investigator triangulation.

Analysis was designed to allow for investigator triangulation. Dr Hamm and the qualitative researchers at Qual EASE are not content specialists in Team Science, and, as such, data collection and subsequent analysis were completed by team members who were naïve to the topic in order to reduce bias. Additionally, secondary coding of the 10 transcripts used to establish intercoder reliability was completed by a trained coder who had not participated in interviewing in order to ensure that someone naïve to the project entirely found the coding categories to be relevant. Thematic results were triangulated with other investigators who are content specialists in order to determine what was common to the existing literature in Team Science, and what was novel to this data set.

## Results

We found a high level of satisfaction across teams in the study, but also hints of underlying tensions that had the potential to escalate into larger problems. The data broke down into the following 7 themes, with representative quotations in Table 2. The first 6 of these themes emerged across interviews with all team members, including leaders, while the last was evident primarily in interviews with leaders.

**Table 2:** Key themes and representative quotations

Theme 1: Collegiality	Theme 2: Communication	Theme 3: Goals	Theme 4: Conflict	Theme 5: Resources	Theme 6: Boundaries	Theme 7: Leadership
*I think it’s the atmosphere, like the collegial atmosphere. I don’t think anyone feels threatened by another person. You know personally I feel I can freely express what I think about certain issues that we’re dealing with.* *What we’ve done well I think is a* […] *natural level of *[…] *mutual respect across each part of this team, I think. But it’s hard to give a specific example* […] *and I think much of it is the intangibles, I guess, in terms of how we, um, respect and communicate with each other.* *We’re all colleagues. We value each other’s work, we value each other’s expertise.*	*I think the one thing that was helpful here was that we have open lines of communications and very frequent [communication] such that it was always very clear about what was being done.* *I think the regular structure of meetings is very helpful. Because it does keep everybody updated and on track. I think we try to be pretty open. So when, you know, as the coordinators or research assistants, you know, bring a question or have a concern, I think everybody tries to be open to their thoughts. ‘Cause, you know, many times they have good insight into what’s going on. And I think they communicate well.*	*I’m not aware of any sort of formal written documentation for who is going to do what. It was sort of implied based on […] background and training. And perhaps individual talks that [the Team Lead] had had with the individuals.* *You know, it would be nice to have […] sort of like an X years plan for, for the group. Right now we don’t have that. We’re just sort of like playing it by ear. You know, whatever we can accomplish this year.*	*The only problems we’ve been trying to solve have been research related. But those are just kind of methodological considerations. But there hasn’t been any, you know, personnel issues or conflicts in that regard.* *I think, you know, I’m not even sure I’d call them disagreements. Different people have different perspectives on what may be the best way to move forward. And then you figure that out. I don’t know that that’s disagreements as much as different people have different perspectives. So it never feels to me that they’re disagreements that need to be resolved.*	*[E]ach of us can only contribute a certain number of hours, so it’s not like […] all of my week is, you know, devoted to this, you know, so […] we’re slower than what we wanted our group to be, you know […] in terms of publications and all that stuff.* *[T]hey can’t provide salary support for co-investigators […] or they can’t include co-investigators at all […]. So he wanted to include me as a mentor, and he wanted some help from me about all the statistical aspects of his study. So, I agreed to be on that grant as a mentor* […] *and worked on his statistical analysis plan and everything.*	*[T]here’s not a lot of whole team communication outside that monthly meeting. It’s mostly me communicating one-on-one with certain individuals. And then if need be, I may pull someone else in, or I may pass information along. But I would say I’m probably the go-between, you know, amongst the team members.*	*I guess I am [the leader]. I guess if there was someone in charge it is me. But I mean I try to make [it] so that, you know, I try to make [it] so that we are fairly equal, you know, in our meetings. So that it’s not like I’m just telling people what to do. It’s more of an open dialogue. And everyone—I want it to be an environment where we all feel comfortable, you know, participating and that sort of thing. But I guess tech—strictly speaking, I, I mean, I hired [Statistician], so I guess I am in charge, you know?*

### Theme 1: Team Members Reported Collegiality and High Levels of Trust

Team members spoke frequently about respecting and trusting their teammates, feeling comfortable speaking up, and believing that their concerns would be taken seriously. This perception was often framed as a result of an overall healthy team environment as well as a strong sense of social connection. In the majority of cases, interviewees connected a trusting team environment to the fact that team members had a preexisting relationship: they had worked together in the past and had faith in one another’s skills and expertise. In a number of interviews, trust and collegiality were attributed to the personality traits and organizational skills of the team leader. Trust, in turn, was linked to a high level of autonomy: team members trusted one another to do their work without oversight.

### Theme 2: Responsiveness to Communication and the Free Flow of Information Within Teams was Highly Valued

Many of the team members interviewed spoke about how communication functioned on their teams. In large part, these comments focused on team meetings, in particular their frequency and the modality used (phone, conference call, face-to-face). Team members expressed appreciation when meetings were frequent enough to move a project forward, when meetings were organized and efficient, and when the modality fit the meeting’s purpose (e.g., need for visual aids, personal connection). They also often spoke about the responsiveness of team members to emails, and the willingness of team members to listen to one another and accept advice, including from more junior team members. In several cases, team members praised their team members’ openness to different disciplinary perspectives and their willingness to seek a deeper understanding from colleagues from other fields. Team members’ perceptions of communication on their teams were, by and large, positive, with only a few individuals expressing frustration about inadequately frequent or proactive communication, and then only in reference to specific instances, not general team dynamics.

### Theme 3: Teams Often Lacked Explicitly Stated, High-Level Group Goals, Relying on the Grant for Direction and Focusing on Immediate Task Goals

When asked if their teams had explicit or documented goals, at least 1 member of each team indicated that they did not. In some cases, team members said that they did not see the need for an explicit articulation of goals because their goals were already clearly stated in the grant or mentoring contract, or because they assumed the PI had discussed goals individually with team members. While more senior members of teams (who likely were involved in the grant) were not troubled by a lack of mission statement, junior members sometimes expressed a desire for more explicit understanding of team goals, structure, and roles. Other team members found short-term team goals (e.g., grants and publications) and individual task goals (e.g., recruitment targets, specific statistical analyses) clear, but long-term goals less so. Several spoke of a desire to have a bigger picture of where the team was headed.

### Theme 4: Team Members did not Include Disagreements About Science or Roles in their Definition of Conflict and Tended to Downplay Conflict Generally

When asked about conflict on their teams, team members generally denied that there was any. Nevertheless, there was evidence in interviews of low-level tensions. These included disagreements over scientific approaches as well as tensions over roles and responsibilities. Disagreements over science were generally recognized as productive and necessary, and were frequently handled by deferring to expertise or seniority on the team, by asking for input from someone with more authority outside of the team, and/or by letting the team leader make the final determination. While scientific disagreements did not appear to create conflict among teams in our study, several participants hinted at how they might cause problems in teams with stronger personalities. Several team members also mentioned tensions regarding roles and responsibilities. For instance, two members of 1 team were unhappy that a third member was not meeting what they construed to be her responsibility, while that team member described feeling pressured to produce work for which she felt inadequately compensated. On another team, 1 member expressed dismay that a teammate had taken over a role she herself had hoped to play, which she attributed to poor communication in both directions. Interestingly, while these tensions were notable enough for team members to bring up during interviews, it was never in response to questions about conflict, suggesting that team members were defining conflict primarily as a matter of personality clashes or open confrontation, which they did not report.

### Theme 5: Teams were Resource-Constrained, with the Potential for Tension and Conflict as a Result

Teams of early-career investigators are funded by relatively small grants. This requires PIs to be creative and resourceful in how they form and maintain their teams, often relying on student workers and colleagues contributing at a small percent effort. This situation has inherent tensions. Lack of resources sometimes meant that team members had to assist in roles that were not formally their own. For instance, in 1 case, a mentor whose field was statistics ended up doing statistical work that fell outside his mentoring role simply because there were insufficient resources on the team to hire a statistician. Moreover, the fact that early-career investigators were not able to afford experienced research staff meant that they often had to hire junior people and provide more training and upskilling than they anticipated. This led to heavier workloads for other team members and project delays. Because some team members worked for low percent effort, moreover, they did not always prioritize the team’s work relative to other professional commitments, which also caused delays.

### Theme 6: Team Membership was Not Always Clear, with Overlapping Groups and Lines of Affiliation Often Running Through the PI

In interviews, we asked each team member to identify the members of his or her team. The answers revealed an interesting phenomenon: individual team members often do not share a common understanding of the team’s boundaries and members. In 1 case, 2 people identified by the PI as team members did not identify one another as teammates. In another case, 1 team member identified a mentor as part of the team, but the PI did not. In a third case, when asked about her team, the PI spent most of her time talking about a team other than the one our research was targeting. In other words, team boundaries were not well defined. Team members often belonged to multiple, overlapping teams, not all team members saw themselves as part of the team, and not all team members knew all the other team members. Often the PI served as a hub for team interactions in a way that limited interaction between team members, which might account for the lack of a clear sense of team boundaries or identity.

### Theme 7: Early-Career Investigators Appeared Uncomfortable Assuming a Leadership Role

PIs we interviewed seemed somewhat uncomfortable with the idea of hierarchy and leadership, tending to describe themselves as only nominally in charge. For example, in response to the question, “Are you in charge?” several team leaders responded equivocally, indicating ambivalence about claiming that role, and in 1 case, a team member expressed doubt that the PI would think of herself as in charge. To some extent, ambivalence about leadership seemed connected to an egalitarian ethic, but it also seemed to reflect a genuine reluctance to assume the mantle of “leader,” a situation exacerbated by the fact that, because of the presence of mentors, PIs were often not the most experienced members of their teams.

## Discussion

That teams who were interviewed perceived high levels of trust and collegiality was consistent with the research literature’s focus on psychological safety as a key component of successful team functioning [4,15]. So, too, was the emphasis on clear, regular communication, which facilitates knowledge integration and the development of shared mental models [16,17]. These are positive signs and may point to the fact that young investigators are getting more early team experiences than previous generations.

However, the data also point to issues that research suggests could inhibit team functioning. These issues include lack of clarity about high-level team goals, the tendency to define conflict narrowly and downplay it, unclear and overlapping group boundaries, discomfort with leadership, and tensions arising from resource limitations. Research on teams holds that articulating high-level team goals is important for sustaining group motivation [13], aligning goals successfully across groups [4,18], and aligning individual with team goals [19]. Thus, leaving goal-setting to grants and individual conversations may not be optimal. The literature also suggests that conflict is important to recognize and address early [20]; thus, the tendency of teams to define conflict narrowly or deny its existence could inhibit appropriate and early intervention. While the literature recognizes the tendency of modern science teams to have fluid boundaries [21] with potential benefits vis-à-vis knowledge sharing and innovation, it also acknowledges that unclear boundaries can erode psychological bonds and make establishing psychological safety more difficult [22]. The SciTS literature does not explicitly address strategies for managing resource limitations on teams, which suggests that this is an area that needs development.

Of particular note in our findings was the uncertainty about leadership expressed by the PIs we interviewed. We see this uncertainty as a double-edged sword. On the one hand, a more egalitarian team dynamic may reduce perceived power differentials, with advantages for psychological safety [10]. On the other hand, the tendency of PIs to conflate leadership with hierarchy and distance themselves from a leadership role may ultimately inhibit their ability to develop a strong leadership identity and the confidence required to set direction and provide motivation [18].

Our data have a number of implications for the development of appropriate team training for early-career investigators. First, team science training might go further in emphasizing the importance of explicitly communicated and collaboratively crafted team goals, even when there is a grant that presumably makes these goals clear. Team leaders should be given tools and strategies to help their team collectively define, refine, and (if necessary) modify group goals, and to make sure team goals are aligned with individual goals.

Second, team training should not only teach teams to prevent and manage outright conflict, it should also provide tools and strategies to help team members navigate subtler tensions regarding roles and expectations. Moreover, in recognition that certain types of conflict (over scientific approach, for example) can be productive, training should offer tools and language to negotiate those differences of opinion successfully.

Third, given that running a team with limited resources is uniquely challenging for early-career investigators, managing this challenge would be an excellent focus for training. Among other things, junior investigators should be encouraged to set realistic expectations for hiring and training and to create cross-team transparency about each individual’s percent effort and the time they have committed to the project.

Fourth, team training for early-career investigators should not assume stable, clearly defined teams, or an established sense of identity among team members. Instead, training should offer strategies investigators can use to enhance a sense of team membership and shared identity.

Finally, team training should not assume that investigators fully “own” the role of leader—or even necessarily recognize themselves in that description. Rather, training should begin with the assumption that assuming leadership is a challenge in and of itself, and help early-career investigators transition into that role. In particular, team training should help trainees distinguish hierarchical from collaborative leadership styles, exploring leadership identities that are consistent with egalitarian values without rejecting leadership itself.

## Limitations

There were several limitations of this study. Given how common teamwork, team training, and leadership training are within and outside academia, we cannot control for all prior experiences that might have influenced study outcomes. The study was conducted at a single institution with a relatively small sample, made even smaller when stratified by team role. Because it was a self-selecting sample, we likely heard more from PIs who assumed their teams were running smoothly than from PIs who suspected dissatisfaction among team members. While the interviewers and qualitative analysts felt that thematic saturation was reached with regard to the experience of being a team member of a team run by an early-career investigator at this institution, we did not have enough interviews with individuals in each role type to feel that thematic saturation on the experiences of, for example, statisticians or research assistants, had been reached. Moreover, given the relatively small sample, the generalizability of these findings to other institutions would need to be tested.

## Conclusions

The SciTS has provided important conceptual grounding on the issues that matter for successful team functioning, and is now poised to explore specific issues in team leadership and team dynamics that impact teams led by researchers at different career stages. This study suggests that the teams of early-career investigators experience unique issues and challenges that are worth understanding separately from general issues facing science teams.

Understanding these challenges is essential for providing appropriate training and support at a critical stage of professional development. This paper represents a step in that direction.

## Supplementary material

For supplementary material accompanying this paper visit https://doi.org/10.1017/cts.2018.335. click here to view supplementary material
