# Supplementary material for: The teams of early-career investigators: A qualitative pilot study
Source: J Clin Transl Sci. 2019 Feb 5;2(5):321–6. doi: 10.1017/cts.2018.335 (PMC6390388; doi:10.1017/cts.2018.335)
Supplement: Supplementary file 1 [file S2059866118003357sup.zip › S2059866118003357sup001.docx]

Table 2: Key Themes and Representative Quotations

| Theme 1: Collegiality | Theme 2: Communication | Theme 3:  Goals | Theme 4:  Conflict | Theme 5: Resources | Theme 6: Boundaries | Theme 7: Leadership |
| --- | --- | --- | --- | --- | --- | --- |
| *I think it’s the atmosphere, like the collegial atmosphere. I don’t think anyone feels threatened by another person. You know personally I feel I can freely express what I think about certain issues that we’re dealing with.*  *What we’ve done well I think is a…natural level of…mutual respect across each part of this team, I think. But it’s hard to give a specific example… and I think much of it is the intangibles, I guess, in terms of how we, um, respect and communicate with each other.*  *We’re all colleagues. We value each other’s work, we value each other’s expertise.* | *I think the one thing that was helpful here was that we have open lines of communications and very frequent [communication] such that it was always very clear about what was being done.*  *I think the regular structure of meetings is very helpful. Because it does keep everybody updated and on track. I think we try to be pretty open. So when, you know, as the coordinators or research assistants, you know, bring a question or have a concern, I think everybody tries to be open to their thoughts. ‘Cause, you know, many times they have good insight into what’s going on. And I think they communicate well* | *I’m not aware of any sort of formal written documentation for who is going to do what. It was sort of implied based on…background and training. And perhaps individual talks that [the Team Lead] had had with the individuals.*  *You know, it would be nice to have…sort of like an X years plan for, for the group. Right now we don’t have that. We’re just sort of like playing it by ear. You know, whatever we can accomplish this year.* | *The only problems we’ve been trying to solve have been research related. But those are just kind of methodological considerations. But there hasn’t been any, you know, personnel issues or conflicts in that regard.*  *I think, you know, I’m not even sure I’d call them disagreements. Different people have different perspectives on what may be the best way to move forward. And then you figure that out. I don’t know that that’s disagreements as much as different people have different perspectives. So it never feels to me that they’re disagreements that need to be resolved* | *[E]ach of us can only contribute a certain number of hours, so it’s not like…all of my week is, you know, devoted to this, you know, so… we’re slower than what we wanted our group to be, you know… in terms of publications and all that stuff.*  *[T]hey can’t provide salary support for co-investigators…or they can’t include co-investigators at all...So he wanted to include me as a mentor, and he wanted some help from me about all the statistical aspects of his study. So, I agreed to be on that grant as a mentor…and worked on his statistical analysis plan and everything.* | *[T]here’s not a lot of whole team communication outside that monthly meeting. It’s mostly me communicating one-on-one with certain individuals. And then if need be, I may pull someone else in, or I may pass information along. But I would say I’m probably the go-between, you know, amongst the team members.* | *I guess I am [the leader]. I guess if there was someone in charge it is me. But I mean I try to make [it] so that, you know, I try to make [it] so that we are fairly equal, you know, in our meetings. So that it’s not like I’m just telling people what to do. It’s more of an open dialogue. And everyone—I want it to be an environment where we all feel comfortable, you know, participating and that sort of thing. But I guess tech—strictly speaking, I, I mean, I hired [Statistician], so I guess I am in charge, you know.* |
