# Supplementary material for: The teams of early-career investigators: A qualitative pilot study
Source: J Clin Transl Sci. 2019 Feb 5;2(5):321–6. doi: 10.1017/cts.2018.335 (PMC6390388; doi:10.1017/cts.2018.335)
Supplement: Supplementary file 1 [file S2059866118003357sup.zip › S2059866118003357sup002.docx]

Table 1. Team Roles and Number of Participants (n=22)

| Role | # of Participants |
| --- | --- |
| Team Lead/PI | 5 |
| Mentor | 5 |
| Consultant/Collaborator | 5 |
| Research Assistant | 4 |
| Statistician | 2 |
| Research Coordinator | 1 |
